# Supplementary material for: The E3 ligases Itch and WWP2 regulate autoimmune neuroinflammation by controlling TH2 to TH17 cell conversion via interleukin-4-STAT5 axis in mice
Source: Nat Commun. 2026 Jan 23;17:952. doi: 10.1038/s41467-025-67665-w (PMC12848126; doi:10.1038/s41467-025-67665-w)
Supplement: Supplementary file 1 — Supplementary Information [file 41467_2025_67665_MOESM1_ESM.pdf]

The E3 ligases Itch and WWP2 regulate autoimmune neuroinflammation by controlling T_H2 to T_H17 cell conversion via interleukin-4-STAT5 axis in mice.

Authors: Mei Zhao[1]*, Chao Zhang[1], Xin Zhang[1,2], Qingdian Mu[1], Qian Li[1], and Yun-Cai Liu[1,2]*

Affiliations:

[1] Institute for Immunology and School of Basic Medical Science, Tsinghua University; Beijing 100084, China.

[2] Tsinghua-Peking Center for Life Sciences, Tsinghua University, Beijing 100084, China.

*Corresponding author. Email: zhaom18@mail.tsinghua.org.cn;

yuncai_liu@mail.tsinghua.edu.cn.

Additional information

The file includes:

Eight figures. **Supplementary Figs. 1 to 8**

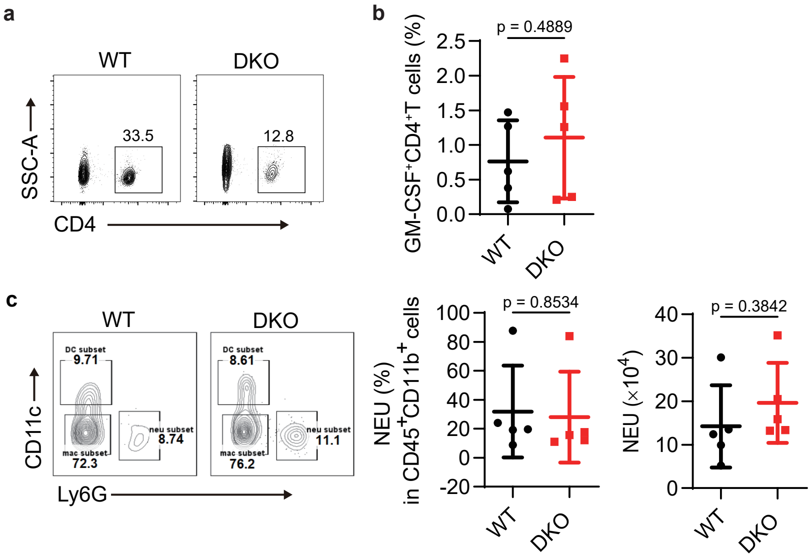
a
WT
DKO
33.5
12.8
SSC-A
CD4
b
p = 0.4889
GM-CSF+CD4+T cells (%)
2.5
2.0
1.5
1.0
0.5
0.0
WT
DKO
c
WT
DKO
DC subset
9.71
8.61
neu subset
8.74
11.1
mac subset
72.3
76.2
CD11c
Ly6G
p = 0.8534
NEU (%)
in CD45+CD11b+ cells
100
80
60
40
20
0
-20
WT
DKO
p = 0.3842
NEU (×10^4)
40
30
20
10
0
WT
DKO

Supplementary Fig. 1 Related to Fig. 1 DKO mice spontaneously develop atypical EAE. a Flow cytometry of splenic $CD4^+$ T cells from 6- to 8-week-old WT and DKO mice. **b** Percentage of GM-CSF production in splenic $CD4^+$ T cells from WT and DKO mice (key; *n*= 5 per group). Data are represented as mean ± SD. *p* value was evaluated by unpaired two-tailed Student's t test. **c** Frequencies and cell numbers of neutrophils (NEU) in the CNS of WT and DKO mice (key; *n*= 5 per group). Data are represented as mean ± SD. *p* values were evaluated by unpaired two-tailed Student's t test. Each symbol represents one individual mice. Data are representative of at least three independent experiments.

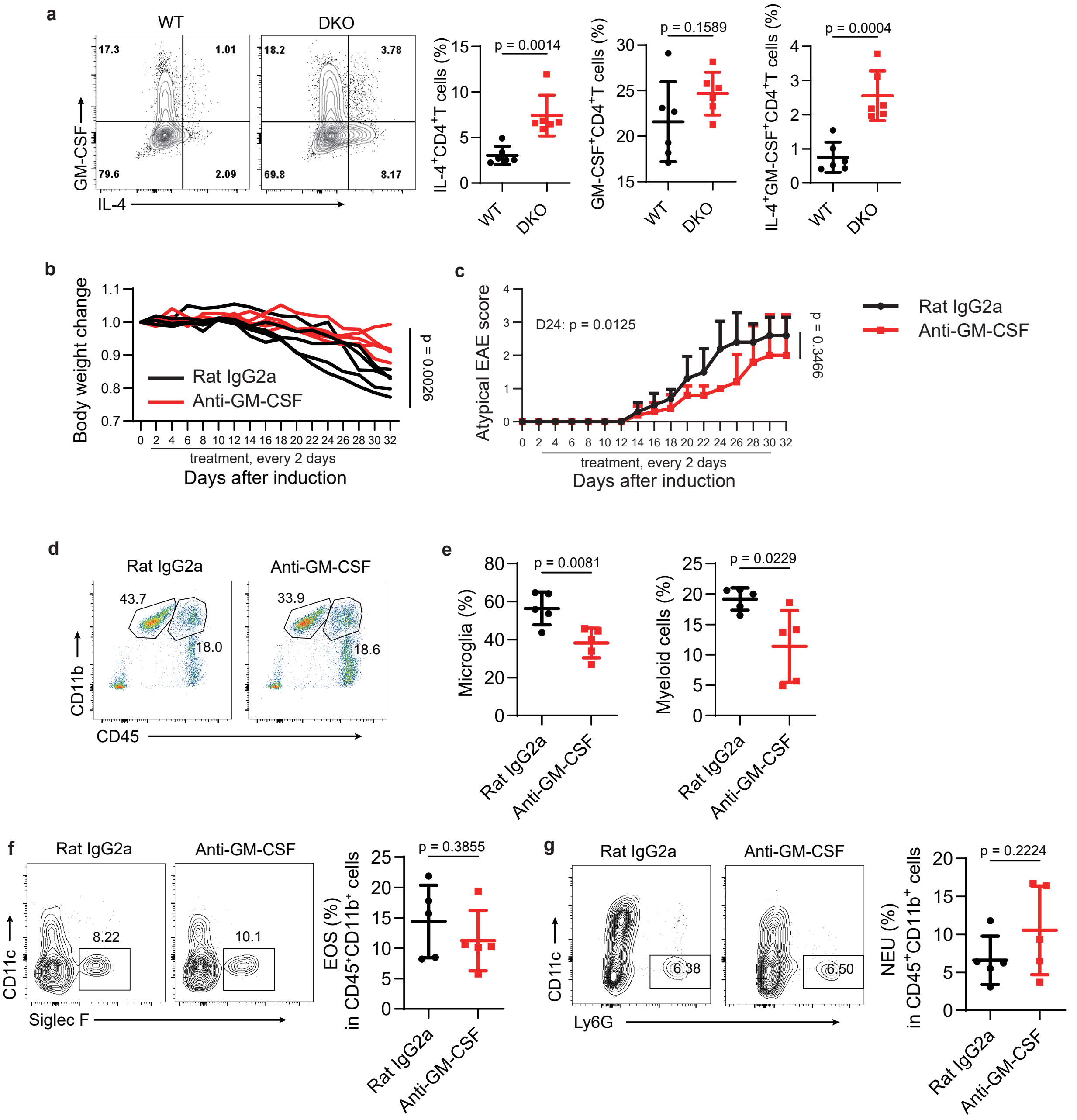
a
WT
DKO
17.3
1.01
79.6
2.09
18.2
3.78
69.8
8.17
GM-CSF
IL-4
IL-4+CD4+T cells (%)
p = 0.0014
GM-CSF+CD4+T cells (%)
p = 0.1589
IL-4+GM-CSF+CD4+T cells (%)
p = 0.0004
b
Body weight change
Rat IgG2a
Anti-GM-CSF
p = 0.0026
treatment, every 2 days
Days after induction
c
Atypical EAE score
D24: p = 0.0125
p = 0.3466
d
Rat IgG2a
Anti-GM-CSF
43.7
18.0
33.9
18.6
CD11b
CD45
e
Microglia (%)
p = 0.0081
Myeloid cells (%)
p = 0.0229
f
8.22
10.1
CD11c
Siglec F
EOS (%) in CD45+CD11b+ cells
p = 0.3855
g
6.38
6.50
Ly6G
NEU (%) in CD45+CD11b+ cells
p = 0.2224

Supplementary Fig. 2 The production of IL-4 and GM-CSF in activated CD4$^+$ T cells and the role of GM-CSF in passive EAE model. a The production of IL-4 and GM-CSF in activated CD4$^+$ T cells (key; *n*= 6 per group). Naïve CD4$^+$ T cells were isolated from spleen and lymph nodes in WT and DKO mice and cultured *in vitro* with anti-CD3 and anti-CD28 for 48h, then stimulated for 4h with PMA and ionomycin in the presence of the protein-transport inhibitor GolgiStop. Data are represented as mean ± SD. *p* values were evaluated by unpaired two-tailed Student's t test. **b** Mouse body weight loss in the passive EAE model, following the transfer of DKO CD4$^+$ T cells and treatment with either anti-GM-CSF or Rat IgG2a isotype control (key; *n*= 5 per group). *p* value on Day32 was evaluated by unpaired two-tailed Student's t test. **c** Atypical EAE scores based on ascending ataxia in passive EAE model as in (**b**) (key; *n*= 5 per group). Data are represented as mean ± SD. *p* value on Day32 was evaluated by unpaired two-tailed Student's t test. **d** Flow cytometry of microglia and myeloid cells in the CNS of passsive EAE model as in (**b**). **e** Frequency of microglia and myeloid cells in the CNS of passive EAE model as in (**b**) (key; *n*= 5 per group). Data are represented as mean ± SD. *p* values were evaluated by unpaired two-tailed Student's t test. **f** Frequency of eosinophils in the CNS of passive EAE model as in (**b**) (key; *n*= 5 per group). Data are represented as mean ± SD. *p* value was evaluated by unpaired two-tailed Student's t test. **g** Frequency of neutrophils in the CNS of passive EAE model as in (**b**) (key; *n*= 5 per group). Data are represented as mean ± SD. *p* value was evaluated by unpaired two-tailed Student's t test. Each symbol (**a**) represents one individual biological replicate. Each symbol (**b, c, e-g**)

represents an individual mouse. Data (**a**) are pooled from six independent biological replicates.

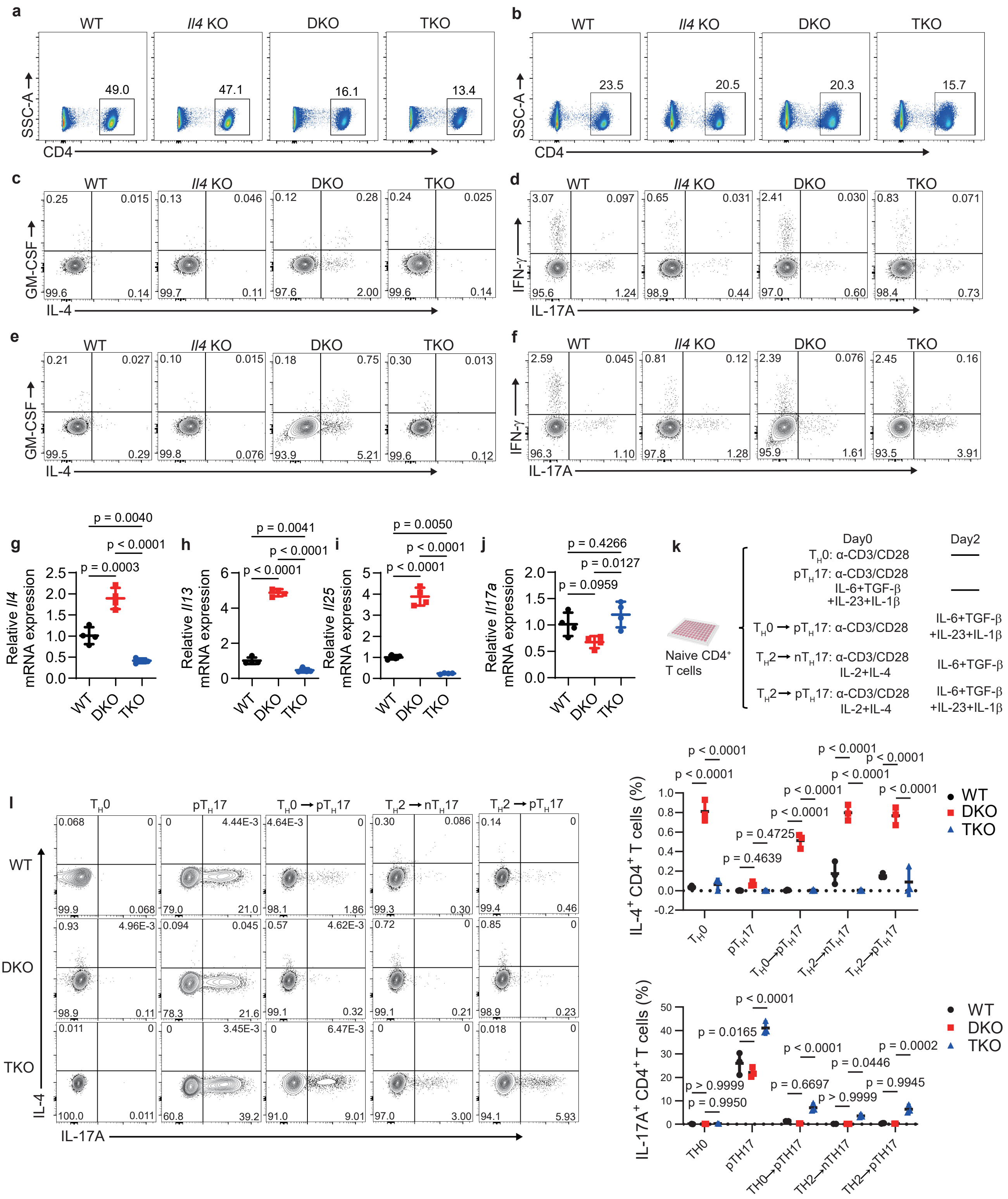
a
WT
Il4 KO
DKO
TKO
49.0
47.1
16.1
13.4
SSC-A
CD4
b
WT
Il4 KO
DKO
TKO
23.5
20.5
20.3
15.7
SSC-A
CD4
c
GM-CSF
IL-4
d
IFN-γ
IL-17A
e
GM-CSF
IL-4
f
IFN-γ
IL-17A
g
Relative Il4 mRNA expression
p = 0.0040
p < 0.0001
p = 0.0003
h
Relative Il13 mRNA expression
p = 0.0041
p < 0.0001
p < 0.0001
i
Relative Il25 mRNA expression
p = 0.0050
p < 0.0001
p < 0.0001
j
Relative Il17a mRNA expression
p = 0.4266
p = 0.0127
p = 0.0959
WT
DKO
TKO
k
Naive CD4+ T cells
Day0
Day2
TH0: α-CD3/CD28
pTH17: α-CD3/CD28 IL-6+TGF-β +IL-23+IL-1β
TH0 → pTH17: α-CD3/CD28
IL-6+TGF-β +IL-23+IL-1β
TH2 → nTH17: α-CD3/CD28 IL-2+IL-4
IL-6+TGF-β
TH2 → pTH17: α-CD3/CD28 IL-2+IL-4
IL-6+TGF-β +IL-23+IL-1β
l
TH0
pTH17
TH0 → pTH17
TH2 → nTH17
TH2 → pTH17
WT
DKO
TKO
IL-4
IL-17A
IL-4+ CD4+ T cells (%)
IL-17A+ CD4+ T cells (%)
WT
DKO
TKO

Supplementary Fig. 3 Assessment of cytokine production of CD4$^+$ T cells from various tissues and in-vitro cultures of WT, *Il4* KO, DKO, and TKO mice. a Flow cytometry of splenic CD4$^+$ T cells from 6- to 7-week-old WT, *Il4* KO, DKO, and TKO mice. **b** Flow cytometry of lung CD4$^+$ T cells from 6- to 8-week-old WT, *Il4* KO, DKO, and TKO mice. **c** Flow cytometry plots illustrating the production of IL-4 and GM-CSF by CD4$^+$ T cells in the lungs of mice described in (**b**). **d** Flow cytometry plots illustrating the production of IL-17A and IFN-γ by CD4$^+$ T cells in the lungs of mice described in (**b**). **e** Flow cytometry plots showing the production of IL-4 and GM-CSF by CD4$^+$ T cells in the lungs of 12-week-old WT, *Il4* KO, DKO, and TKO mice. **f** Flow cytometry plots showing the production of IL-17A and IFN-γ by CD4$^+$ T cells in the lungs of 12-week-old WT, *Il4* KO, DKO, and TKO mice. **g** to **j** Relative mRNA expression levels of *Il4* (**g**), *Il13* (**h**), *Il25* (**i**), *Il17a* (**j**) in the postauricular skin of mice as in (**e**) (key; n= 4 per group). Error bars represent the mean ± SD. *p* values were evaluated by one-way ANOVA with Tukey's test adjusted for multiple comparisons. **k** The procedure for in-vitro T cell differentiation. **l** Frequency of IL-4 and IL-17A production in the CD4$^+$ T cells from WT, DKO, and TKO mice, which were cultured *in vitro* as in (**k**) (key; n= 3 per group). Error bars represent the mean ± SD. *p* values were evaluated by two-way ANOVA with Tukey's test adjusted for multiple comparisons. Each symbol (**g** to **j**) represents an individual mouse. Each symbol (**l**) represents one individual biological replicate. Data are representative of three (**a** to **d**) and four (**e-j**) independent experiments. Data (**l**) are pooled from three independent biological replicates.

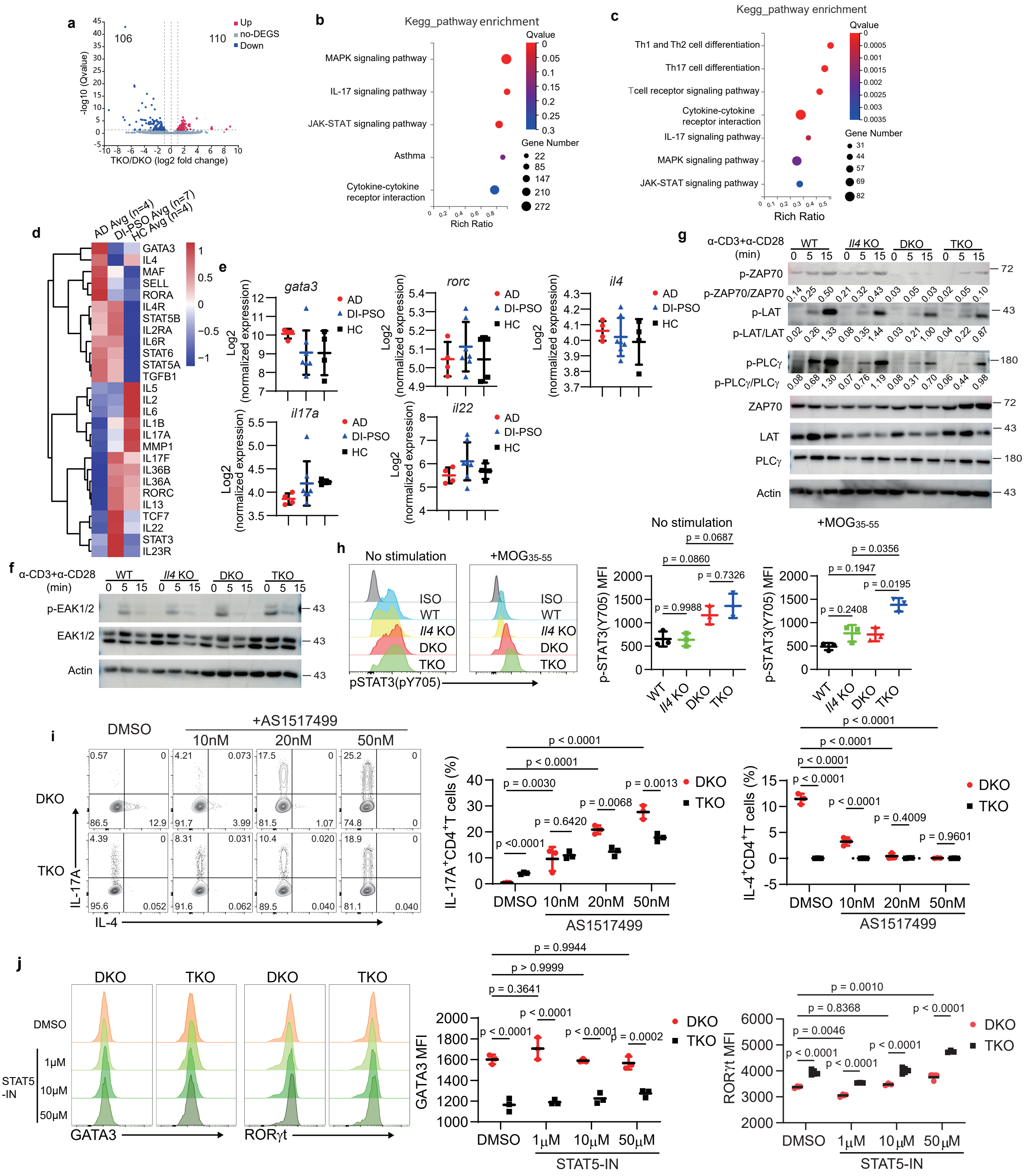

a
Up
no-DEGS
Down
106
110
-log10 (Qvalue)
TKO/DKO (log2 fold change)
b
Kegg_pathway enrichment
MAPK signaling pathway
IL-17 signaling pathway
JAK-STAT signaling pathway
Asthma
Cytokine-cytokine receptor interaction
Qvalue
Gene Number
Rich Ratio
c
Kegg_pathway enrichment
Th1 and Th2 cell differentiation
Th17 cell differentiation
Tcell receptor signaling pathway
Cytokine-cytokine receptor interaction
IL-17 signaling pathway
MAPK signaling pathway
JAK-STAT signaling pathway
Qvalue
Gene Number
Rich Ratio
d
AD Avg (n=4)
DI-PSO Avg (n=7)
HC Avg (n=4)
GATA3 IL4 MAF SELL RORA IL4R STAT5B IL2RA IL6R STAT6 STAT5A TGFB1 IL5 IL2 IL6 IL1B IL17A MMP1 IL17F IL36B IL36A RORC IL13 TCF7 IL22 STAT3 IL23R
e
gata3
rorc
il4
il17a
il22
AD
DI-PSO
HC
Log2 (normalized expression)
f
α-CD3+α-CD28 (min)
WT
Il4 KO
DKO
TKO
p-EAK1/2
EAK1/2
Actin
g
α-CD3+α-CD28 (min)
WT
Il4 KO
DKO
TKO
p-ZAP70
p-ZAP70/ZAP70
p-LAT
p-LAT/LAT
p-PLCγ
p-PLCγ/PLCγ
ZAP70
LAT
PLCγ
Actin
h
No stimulation
+MOG35-55
ISO
WT
Il4 KO
DKO
TKO
pSTAT3(pY705)
p-STAT3(Y705) MFI
i
DMSO
+AS1517499
10nM
20nM
50nM
DKO
TKO
IL-17A
IL-4
IL-17A+CD4+T cells (%)
IL-4+CD4+T cells (%)
AS1517499
j
DKO
TKO
DMSO
STAT5-IN
1μM
10μM
50μM
GATA3
RORγt
GATA3 MFI
RORγt MFI

Supplementary Fig. 4 Related to Fig. 6 IL-4 controls STAT5 phosphorylation and T_H17 cell differentiation. a Differential gene-expression profiles (*x*-axis, log2 (fold change)) and significance (*y*-axis, –log10 (Qvalue)) between DKO and TKO naïve $CD4^+$ T cells. **b**, **c** KEGG-pathway enrichment analysis of the differentially expression genes between naïve **(b)** and polarized **(c)** $CD4^+$ T cells as in (**Fig. 5a**) from TKO and DKO mice. **d**, **e** Comparison of the gene-expression profiles in transcriptomes of dupilumab-induced psoriatic eruption (DI-Pso, n=7 per group), atopic dermatitis (AD, n=4 per group), with those of health controls (HC, n=4 per group) from public data. Each symbol represents an individual person. Error bars represent the mean ± SD. **f**, **g** Immunoblot analysis of phosphorylated and total ERK1/2 **(f)**, ZAP70 **(g)**, LAT **(g)** and PLCγ1 **(g)**, in lysates of $CD4^+$ T cells sorted from WT, *Il4* KO, DKO, and TKO mice and stimulated for 0, 5 or 15 min with anti-CD3 plus anti-CD28. **h** Phosphorylation level of STAT3 in splenocytes as in (**Fig. 6f)** (key; $n = 3$ per group). Error bars represent the mean ± SD. p values were evaluated by one-way ANOVA with Tukey's test adjusted for multiple comparisons. **i** Frequency of IL-4 and IL-17A production in $CD4^+$ T cells from DKO and TKO mice cultured *in vitro* under T_H0 to T_H17 condition with the treatment of AS1517499 (key; $n = 3$ per group). Error bars represent the mean ± SD. p values were evaluated by two-way ANOVA with Tukey's test adjusted for multiple comparisons. **j** Expression of RORγt and GATA3 in $CD4^+$ T cells treated with STAT5-IN as in (**Fig. 6h)** (key; $n = 3$ per group). Error bars represent the mean ± SD. p values were evaluated by two-way ANOVA with Tukey's test adjusted for multiple comparisons. Each symbol (**h-j**) represents one individual biological replicate. Data are pooled from two (**a-c**) and three **(h-j)** independent biological replicates**.** Data (**g, f**) are representative of three independent experiments.

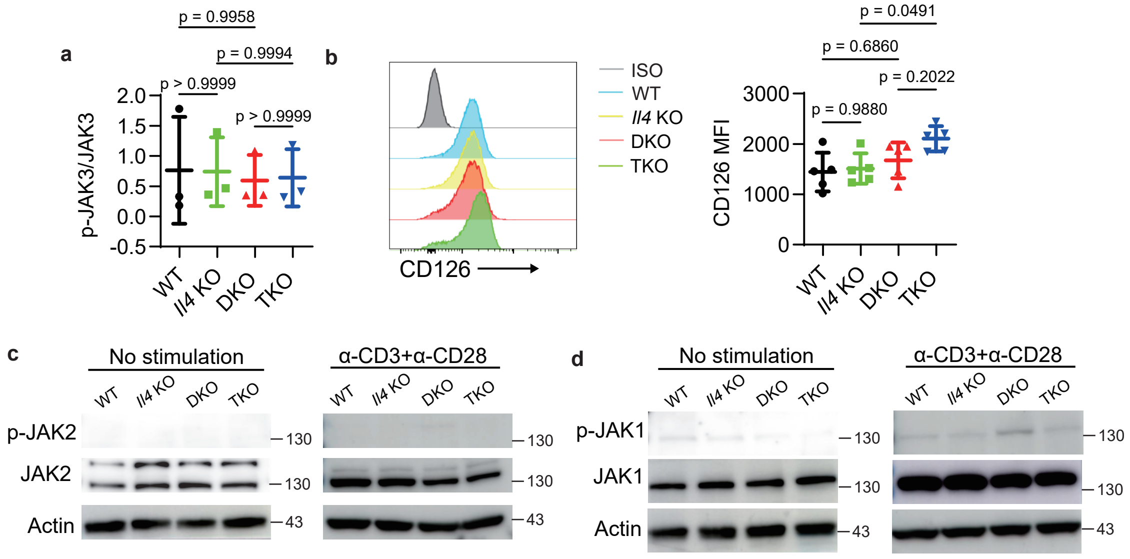
a
p = 0.9958
p = 0.9994
p > 0.9999
p > 0.9999
p-JAK3/JAK3
2.0
1.5
1.0
0.5
0.0
-0.5
WT
Il4 KO
DKO
TKO
b
ISO
WT
Il4 KO
DKO
TKO
CD126
p = 0.0491
p = 0.6860
p = 0.2022
p = 0.9880
CD126 MFI
3000
2000
1000
0
WT
Il4 KO
DKO
TKO
c
No stimulation
α-CD3+α-CD28
WT
Il4 KO
DKO
TKO
p-JAK2
JAK2
Actin
130
130
43
d
No stimulation
α-CD3+α-CD28
WT
Il4 KO
DKO
TKO
p-JAK1
JAK1
Actin
130
130
43

Supplementary Fig. 5 Related to Fig. 7 IL-4R/JAK3/STAT5 signaling pathway controls T_H17 cell differentiation. a Ratios of p-JAK3 and total JAK3 quantifications in lysates of CD4$^+$ T cells as in (**Fig. 7d)** (key; *n*= 3 per group). Error bars represent the mean ± SD. *p* values were evaluated by one-way ANOVA with Tukey's test adjusted for multiple comparisons. **b** Expression of CD126 on splenic CD4$^+$ T cells from mice as in (**Fig. 7a)** (key; *n*= 5 per group). Error bars represent the mean ± SD. *p* values were evaluated by one-way ANOVA with Tukey's test adjusted for multiple comparisons. **c, d** Immunoblot analysis of phosphorylated and total JAK1 **(c)** or JAK2 **(d)**, in lysates of CD4$^+$ T cells sorted from mice as in (**Fig. 7a)** and stimulated for 20 hours with anti-CD3 plus anti-CD28. Each symbol (**a**) represents one individual biological replicate. Each symbol (**b**) represents an individual mouse. Data are pooled from three (**a**) independent biological replicates. Data (**b, c, d**) are representative of at least three independent experiments.

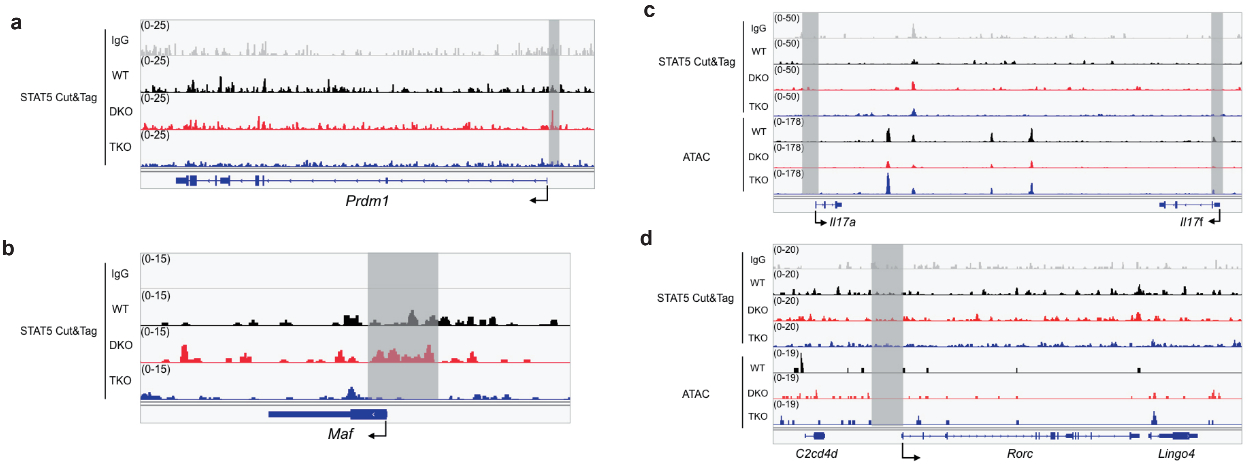
a
(0-25)
IgG
WT
DKO
TKO
STAT5 Cut&Tag
Prdm1
b
(0-15)
IgG
WT
DKO
TKO
STAT5 Cut&Tag
Maf
c
(0-50)
(0-178)
IgG
WT
DKO
TKO
STAT5 Cut&Tag
ATAC
Il17a
Il17f
d
(0-20)
(0-19)
IgG
WT
DKO
TKO
STAT5 Cut&Tag
ATAC
C2cd4d
Rorc
Lingo4

Supplementary Fig. 6 Related to Fig. 8 Stat5 directly binds to *Prdm1* and *Maf* promoters and inhibits T_H17 differentiation in T_H2 environment. a-d STAT5 CUT&Tag track showing STAT5 binding at the *Prdm1* **(a)**, *Maf* **(b)**, *Il17a* **(c)**, *and Rorc* **(d)** gene in WT, DKO, and TKO $CD4^+$ T cells cultured *in vitro* under T_H0 to T_H17 conditions.

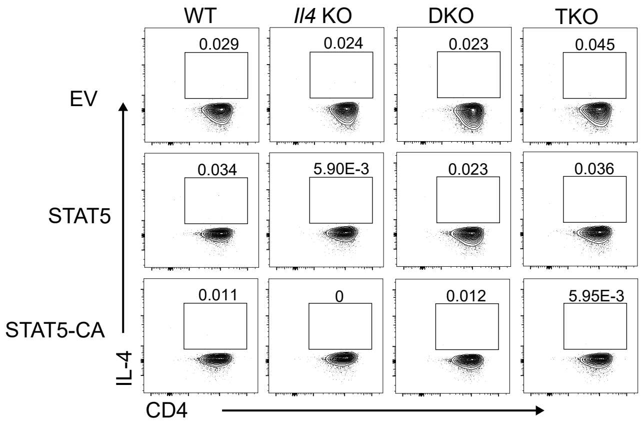

WT
Il4 KO
DKO
TKO
EV
STAT5
STAT5-CA
0.029
0.024
0.023
0.045
0.034
5.90E-3
0.023
0.036
0.011
0
0.012
5.95E-3
IL-4
CD4

Supplementary Fig. 7 Production of IL-4 in STAT5-CA overexpressing CD4$^+$ T cells *in vitro*. Flow cytometry plots illustrating the production of IL-4 in CD4$^+$ T cells, which were infected with STAT5 or a STAT5 mutation as in (**Fig. 8h)**. Data are representative of two independent experiments.

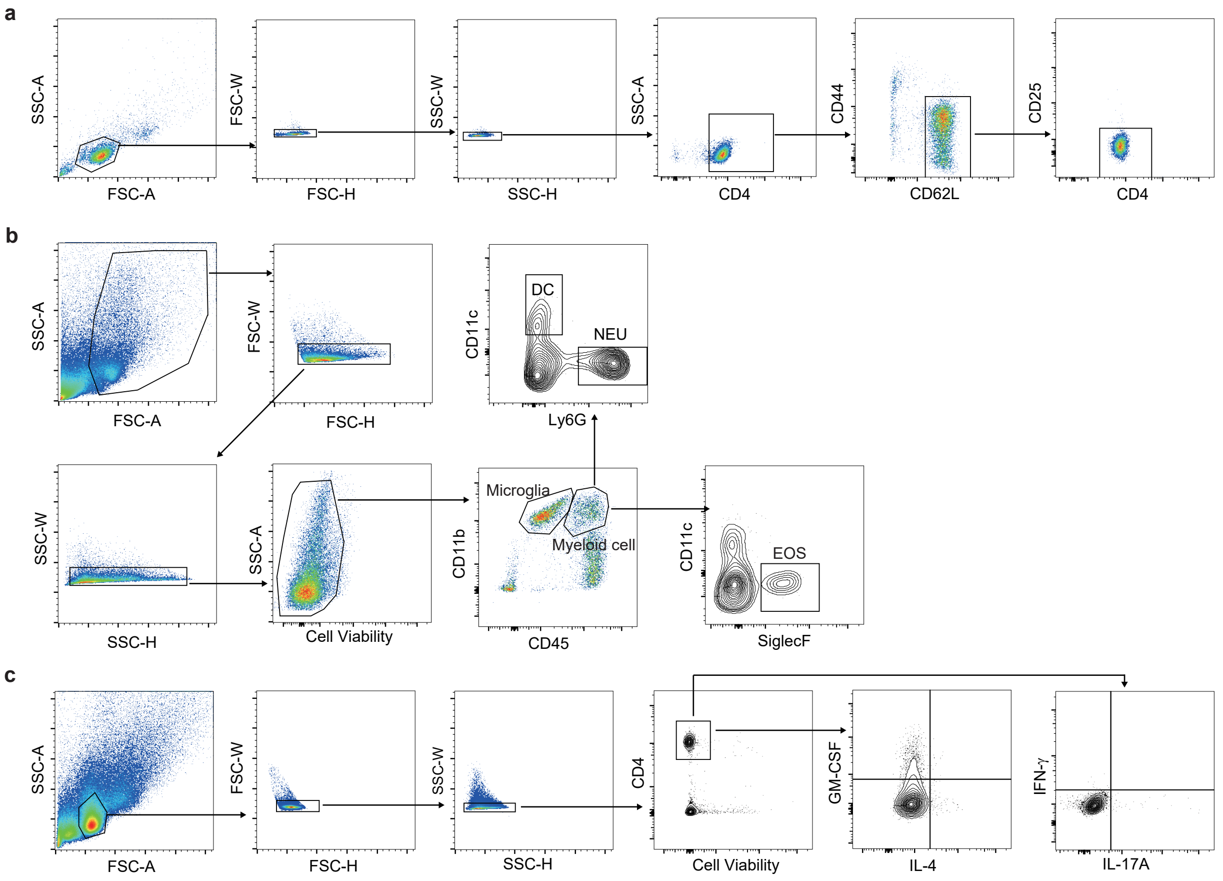
a
SSC-A
FSC-A
FSC-W
FSC-H
SSC-W
SSC-H
SSC-A
CD4
CD44
CD62L
CD25
CD4
b
SSC-A
FSC-A
FSC-W
FSC-H
CD11c
DC
NEU
Ly6G
SSC-W
SSC-H
SSC-A
Cell Viability
Microglia
Myeloid cell
CD11b
CD45
CD11c
EOS
SiglecF
c
SSC-A
FSC-A
FSC-W
FSC-H
SSC-W
SSC-H
CD4
Cell Viability
GM-CSF
IL-4
IFN-γ
IL-17A

Supplementary Fig. 8 Gating strategy. a Sorting strategy for naïve CD4^{+} T cells in the *in vitro* cultures and the passive EAE model. The gating strategy described above applies to Fig. 1e, Fig. 3e, and Fig. 6b. **b** Gating strategy for microglia (CD45^{mid} CD11b^{+}), myeloid cells (CD45^{hi} CD11b^{+}), neutrophils (CD45^{hi} CD11b^{+} Ly6G^{+} CD11c^{-}), dendritic cells (CD45^{hi} CD11b^{+} Ly6G^{-} CD11c^{+}), and eosinophils (CD45^{hi} CD11b^{+} SiglecF^{+} CD11c^{-}). The gating strategy described above applies to Fig. 1j to m, supplementary Fig. 1d, and supplementary Fig. 2d to j. **c** Gating strategy for the analysis of surface marker, cytokine production, phosph-STAT proteins, and transcription factor expression in CD4^{+} T cells. The gating strategy described above applies to Fig. 1d, f; Fig. 2a to c, g, h; Fig. 3d, i, j, k; Fig. 4; Fig. 5f to h; Fig. 6a, c, e, f; Fig. 7f, g, i, m; supplementary Fig. 1a and b; supplementary Fig. 2a; supplementary Fig. 3a to f, k, l, n; supplementary Fig. 4h to j; supplementary Fig. 5a, b; supplementary Fig. 7.
