# Supplementary material for: The E3 ligases Itch and WWP2 regulate autoimmune neuroinflammation by controlling TH2 to TH17 cell conversion via interleukin-4-STAT5 axis in mice
Source: Nat Commun. 2026 Jan 23;17:952. doi: 10.1038/s41467-025-67665-w (PMC12848126; doi:10.1038/s41467-025-67665-w)
Supplement: Supplementary file 3 — Reporting Summary [file 41467_2025_67665_MOESM3_ESM.pdf]

nature portfolio

Corresponding author(s): Yun-Cai Liu, Mei Zhao

Last updated by author(s): Nov 21, 2025

Reporting Summary

Nature Portfolio wishes to improve the reproducibility of the work that we publish. This form provides structure for consistency and transparency in reporting. For further information on Nature Portfolio policies, see our Editorial Policies and the Editorial Policy Checklist.

Statistics

For all statistical analyses, confirm that the following items are present in the figure legend, table legend, main text, or Methods section.

n/a	Confirmed	
☐	☒	The exact sample size (*n*) for each experimental group/condition, given as a discrete number and unit of measurement
☐	☒	A statement on whether measurements were taken from distinct samples or whether the same sample was measured repeatedly
☐	☒	The statistical test(s) used AND whether they are one- or two-sided<br>*Only common tests should be described solely by name; describe more complex techniques in the Methods section.*
☒	☐	A description of all covariates tested
☐	☒	A description of any assumptions or corrections, such as tests of normality and adjustment for multiple comparisons
☐	☒	A full description of the statistical parameters including central tendency (e.g. means) or other basic estimates (e.g. regression coefficient) AND variation (e.g. standard deviation) or associated estimates of uncertainty (e.g. confidence intervals)
☐	☒	For null hypothesis testing, the test statistic (e.g. *F*, *t*, *r*) with confidence intervals, effect sizes, degrees of freedom and *P* value noted<br>*Give P values as exact values whenever suitable.*
☒	☐	For Bayesian analysis, information on the choice of priors and Markov chain Monte Carlo settings
☒	☐	For hierarchical and complex designs, identification of the appropriate level for tests and full reporting of outcomes
☒	☐	Estimates of effect sizes (e.g. Cohen's *d*, Pearson's *r*), indicating how they were calculated

Our web collection on statistics for biologists contains articles on many of the points above.

Software and code

Policy information about availability of computer code

Data collection	No software was used for data collection.
Data analysis	R (4.1.0)<br>Dr.Tom (v.2.0)<br>Trimmomatic (v.0.32)<br>Bowtie 2 (v.2.4.2)<br>Samtools (v.1.11)<br>Macs2 (v.2.1.0)<br>Deeptools (v.3.5.4)<br>DiffBind (v.3.12.0)<br>Bioconductor-CHIP seeker (v.1.28.3)<br>IGV (v.2.11.9)<br>GSEA (v.4.4.0)<br>Transcriptome Analysis Console (v.4.0)<br>FlowJo (v.10)<br>GraphPad Prism (v.10)<br>ImageJ (v.1.54g)<br>ZEN (v.3.1)

For manuscripts utilizing custom algorithms or software that are central to the research but not yet described in published literature, software must be made available to editors and reviewers. We strongly encourage code deposition in a community repository (e.g. GitHub). See the Nature Portfolio guidelines for submitting code & software for further information.

Data

Policy information about availability of data

All manuscripts must include a data availability statement. This statement should provide the following information, where applicable:

- Accession codes, unique identifiers, or web links for publicly available datasets
- A description of any restrictions on data availability
- For clinical datasets or third party data, please ensure that the statement adheres to our policy

Data associated with all figures and tables is provided in the Source data file and Supplementary files. The raw sequence data reported in this paper have been deposited in the Genome Sequence Archive at National Genomics Data Center, China National Center for Bioinformation / Beijing Institute of Genomics, Chinese Academy of Sciences (https://ngdc.cncb.ac.cn/gsa/search?searchTerm=CRA027859, accession code CRA027859). Previously published human patients skin microarray datasets used in this study is available in the Gene Expression Omnibus database (https://www.ncbi.nlm.nih.gov/geo/query/acc.cgi?acc=GSE185764, accession code GSE185764). Source data are provided with this paper.

Research involving human participants, their data, or biological material

Policy information about studies with human participants or human data. See also policy information about sex, gender (identity/presentation), and sexual orientation and race, ethnicity and racism.

Reporting on sex and gender	N/A
Reporting on race, ethnicity, or other socially relevant groupings	N/A
Population characteristics	N/A
Recruitment	N/A
Ethics oversight	N/A

Note that full information on the approval of the study protocol must also be provided in the manuscript.

Field-specific reporting

Please select the one below that is the best fit for your research. If you are not sure, read the appropriate sections before making your selection.

☒ Life sciences ☐ Behavioural & social sciences ☐ Ecological, evolutionary & environmental sciences

For a reference copy of the document with all sections, see nature.com/documents/nr-reporting-summary-flat.pdf

Life sciences study design

All studies must disclose on these points even when the disclosure is negative.

Sample size	No sample size calculation was performed. Sample size was based on the experimental aim and availability of age-matched mice with genotype difference in most experiments. Based on our previous studies in characterization of CD4 T cell function (Daisuke etal., Nat Immunol,<br>2018), we estimated that a sample size of 3-10 was sufficient to detect statistically significant difference between groups. The number of tested sample and independent experiment were indicated in each figure legend.
Data exclusions	No data was excluded from analyses.
Replication	All experiments were performed at least three independent times under identical conditions, unless otherwise stated. The replicate number is always clearly stated in the legends.
Randomization	No randomization was used in the experiments. Experimental groups were based on genotypes of age-matched mice in each experiment.
Blinding	The investigators were not blinded during experiments and data analysis, since most of experimental results were consistent irrespective of blindness. All conclusions in this study were made based on statistical significance of the data.

Reporting for specific materials, systems and methods

We require information from authors about some types of materials, experimental systems and methods used in many studies. Here, indicate whether each material, system or method listed is relevant to your study. If you are not sure if a list item applies to your research, read the appropriate section before selecting a response.

Materials & experimental systems

n/a	Involved in the study
☐	☒ Antibodies
☐	☒ Eukaryotic cell lines
☒	☐ Palaeontology and archaeology
☐	☒ Animals and other organisms
☒	☐ Clinical data
☒	☐ Dual use research of concern
☒	☐ Plants

Methods

n/a	Involved in the study
☐	☒ ChIP-seq
☐	☒ Flow cytometry
☒	☐ MRI-based neuroimaging

Antibodies

Antibodies used	For T cell culture, antibodies to mouse CD3ε (clone 145-2C11, #BE0001-1, 3 μg/ml), CD28 (clone 37.51, #BE0015-1, 2 μg/ml), IFNγ (clone R4-6A2, #BE0054, 10 μg/ml), IL-2(clone JES6-1A12, # BE0043, 20ng/ml) were from Bio X Cell; purified anti-mouse CD25 antibody were from Bio Legend (clone 3C7, # 101902, 1 μg/ml). For flow cytometry and ELISA, anti-mouse CD44-FITC (clone IM7, #45-0441-82, 1:500), anti-mouse Blimp-1-PE (5E7, #150005, 1:100), anti-mouse/human CD11b-BV421 (clone M1/70, #101236, 1:500), anti-mouse CD11c-PE/Cy7 (clone N418, #117318, 1:500), anti-mouse Ly6G/Ly6C(Gr1)-AF700 (clone 1A8, #108422, 1:500), Purified anti-mouse IgE (clone RME-1, #406902, 1:2000), Purified anti-mouse IgG1 (clone RMG1-1, #406602, 1:4000) were from Bio Legend; anti-mouse CD4-eF450 (clone RM4-5,#48-0042-82, 1:500), anti-mouse CD25-PE (PC61.5, #25-0251-82, 1:500), anti-mouse CD62L-PE/Cy7 (clone MEL-14, # 25-0621-82, 1:500), anti-mouse CD126-PE (clone D7715A7,# 12-1261-80, 1:500), Anti-Mouse/Rat IL-17A-PE (clone 17B7, #12-7177-81, 1:400), anti-mouse IFN-γ-eF450 (clone XMG1.2, # 48-7311-82, 1:200), anti-mouse GM-CSF-PE/Cy7 (clone MP1-22E9, #25-7331-82, 1:100), anti-mouse RORγt-PerCP-Cy5.5 (clone B2D, #46698182, 1:100), anti-mouse/human c-Maf-PE (clone sym0F1, # 12-9855-41, 1:100) were from eBioscience; and anti-mouse Siglec-F-PE-CF594 (clone E50-2440, #562757, 1:1000), anti-mouse CD124-PE (clone mIL4R-M1, #552509, 1:500), anti-mouse IL-4-FITC (clone11B11, #557728, 1:100), anti-mouse GATA-3-FITC (clone L50-823, #560163, 1:100) were obtained from BD Biosciences. For Phosflow analysis, anti-mouse/human pSTAT3 at Tyr705-PE (eBioscience, clone LUVNKLA, #12-9033-42, 1:100), anti-mouse pSTAT5 at TyrY649-APC (BD Biosciences, clone 47, #612599, 1:100), anti-mouse pSTAT6 at TyrY641-PE (BD Biosciences, clone J71-773.58.11, #558252, 1:100). For immunoblot analysis, antibody to mouse Lck (clone 3A5, #sc-433, 1:2000) was from Santa Cruz Biotechnology; antibody to mouse ZAP70/Syk phosphorylated at Tyr319 and Tyr352 (clone 65E4, #2717, 1:1000), ZAP70 (clone D1C10E, #3165, 1:2000), PLCγ1 phosphorylated at Tyr783 (#2821, 1:1000), PLCγ1 (clone D9H10, #5690, 1:2000), Jak1 phosphorylated at Tyr1034 and Tyr1035 (clone D7N4Z, #74129, 1:1000), Jak1 (clone D1T6W, #50996, 1:2000), Jak2 phosphorylated at Tyr1008 (clone D4A8, #8082, 1:1000), Jak2 (clone D2E12, #3230, 1:2000), Jak3 phosphorylated at Tyr980 and Tyr981 (clone D44E3, #5031, 1:1000), Jak3 (clone D7B12, #8863, 1:2000), p44/42 MAPK (Erk1/2) phosphorylated at Thr202 and Tyr204 (clone D13.14.4E, #4370, 1:1000), p44/42 MAPK (Erk1/2) (clone 137F5, #4695, 1:2000) were from Cell Signaling Technology; antibodies to mouse LAT phosphorylated at Tyr191 (#07-278, 1:1000) and LAT (#06-807, 1:2000) were from Millipore; antibodies to Actin (clone AC-74, #A2228, 1:5000) was from Sigma-Aldrich; antibody to mouse Lck phosphorylated at Tyr394 (#PA5-37628, 1:1000) was from Thermo Fisher Scientific. For CUT&Tag analysis, antibody to mouse STAT5(D2O6Y, #94205, 1:50) was from Cell Signaling Technology; antibody to Rabbit IgG H&L(#ab6702, 1:100) was from Abcam. For anti-GM-CSF treatment, antibody to mouse GM-CSF (clone MP1-22E9, #HY-P99134, 20mg/kg) and isotype control Rat IgG2a kappa (#HY-P990679, 20mg/kg) were from MCE.
Validation	All antibodies used in this study were obtained from commercial sources and validated according to manufacturers's instruction.

Eukaryotic cell lines

Policy information about cell lines and Sex and Gender in Research

Cell line source(s)	HEK293T cells, Platinum-Eco cells
Authentication	The cell lines were not authenticated.
Mycoplasma contamination	All cell lines were tested Mycoplasma negative.
Commonly misidentified lines (See ICLAC register)	No commonly misidentified cell lines were used in this study.

Animals and other research organisms

Policy information about studies involving animals; ARRIVE guidelines recommended for reporting animal research, and Sex and Gender in Research

Laboratory animals	The 2D2 TCR transgenic mice and CD4-cre mice were kindly provided by Professor Chen Dong of Tsinghua University, Beijing, China. Il4−/− mice and Rag1−/− mice were acquired from the Jackson Laboratory. The floxed Itch mice on a B6 background were generated by using a standard gene-targeting strategy with help from A. Tarakhvosky. Wwp2-/- mice were provided by Professor Dallas Jones. All mice used in the experiments were on 2D2 TCR transgenic background. Both male and female mice were used in experiments.

Mice were used between 6-20 weeks of age. The mice were maintained under specific pathogen-free (SPF) conditions at Laboratory Animal Resources Center, Tsinghua University. The dark/light cycle is 12 hours/12 hours in the 24-hour cycle. The ambient temperature is 23-26?, and humidity is 40%-70%.
All studies were approved by the Animal Care and Use Committee of Tsinghua University.

Wild animals

The study did not involve wild animals.

Reporting on sex

6–8 week old male and female mice were examined in this study, with similar findings observed for both sexes. Female Rag1−/− mice were utilized as recipient mice due to their increased susceptibility to EAE. Nevertheless, our findings are expected to be relevant to both sexes, since males develop EAE with a similar physiopathology.

Field-collected samples

The study did not involve samples collected from the field.

Ethics oversight

All studies were approved by the Animal Care and Use Committee of Tsinghua University, Beijing.

Note that full information on the approval of the study protocol must also be provided in the manuscript.

Plants

Seed stocks

N/A

Novel plant genotypes

N/A

Authentication

N/A

ChIP-seq

Data deposition

☒ Confirm that both raw and final processed data have been deposited in a public database such as GEO.

☐ Confirm that you have deposited or provided access to graph files (e.g. BED files) for the called peaks.

Data access links
May remain private before publication.

https://ngdc.cncb.ac.cn/gsa/search?searchTerm=CRA027859

Files in database submission

RNA seq:
Spleen_CD4T_WT_1.fq.gz
Spleen_CD4T_WT_2.fq.gz
Spleen_CD4T_Wwp2_Itch_dKO_1.fq.gz
Spleen_CD4T_Wwp2_Itch_dKO_2.fq.gz
Spleen_CD4T_Wwp2_Itch_dKO_3.fq.gz
Spleen_CD4T_Wwp2_Itch_Il4_tKO_1.fq.gz
Spleen_CD4T_Wwp2_Itch_Il4_tKO_2.fq.gz
Spleen_CD4T_Wwp2_Itch_Il4_tKO_3.fq.gz
Spleen_CD4T_Il4_KO_1.fq.gz
Spleen_CD4T_Il4_KO_2.fq.gz
Spleen_CD4T_Il4_KO_3.fq.gz
InVitro_CD4T_WT_1.fq.gz
InVitro_CD4T_WT_2.fq.gz
InVitro_CD4T_Wwp2_Itch_dKO_1.fq.gz
InVitro_CD4T_Wwp2_Itch_dKO_2.fq.gz
InVitro_CD4T_Wwp2_Itch_Il4_tKO_1.fq.gz
InVitro_CD4T_Wwp2_Itch_Il4_tKO_2.fq.gz
InVitro_CD4T_Il4_KO_1.fq.gz
InVitro_CD4T_Il4_KO_2.fq.gz
ATAC-seq:
CD4T_ATAC_WT_R1.fq.gz
CD4T_ATAC_WT_R2.fq.gz
CD4T_ATAC_Wwp2_Itch_dKO_R1.fq.gz
CD4T_ATAC_Wwp2_Itch_dKO_R2.fq.gz
CD4T_ATAC_Wwp2_Itch_Il4_tKO_R1.fq.gz
CD4T_ATAC_Wwp2_Itch_Il4_tKO_R2.fq.gz
CD4T_ATAC_Il4_KO_R1.fq.gz
CD4T_ATAC_Il4_KO_R2.fq.gz
CUT&Tag:
CD4T_cut_tag_WT_stat5_R1.fastq.gz

CD4T_cut_tag_WT_stat5_R2.fastq.gz
CD4T_cut_tag_Wwp2_Itch_dKO_stat5_R1.fastq.gz
CD4T_cut_tag_Wwp2_Itch_dKO_stat5_R2.fastq.gz
CD4T_cut_tag_Wwp2_Itch_Il4_tKO_stat5_R1.fastq.gz
CD4T_cut_tag_Wwp2_Itch_Il4_tKO_stat5_R2.fastq.gz
CD4T_cut_tag_Il4_KO_stat5_R1.fastq.gz
CD4T_cut_tag_Il4_KO_stat5_R2.fastq.gz
CD4T_cut_tag_IgG_Control_R1.fastq.gz
CD4T_cut_tag_IgG_Control_R2.fastq.gz

Genome browser session (e.g. UCSC): *Provide a link to an anonymized genome browser session for "Initial submission" and "Revised version" documents only, to enable peer review. Write "no longer applicable" for "Final submission" documents.*

Methodology

Replicates: Biological duplicates/triplicates for RNA-seq samples; and one sequence sample and corresponding input or control sample for ATAC-seq and CUT&Tag samples.

Sequencing depth: 5-30 million raw reads were sequenced per sample.

Antibodies: anti-STAT5 (D2O6Y) antibody(94205, Cell Signaling); Anti-rabbit-IgG (AB6702, Abcam);

Peak calling parameters: Transcription factor peaks were called using MACS2 with -p 1e-5 while adding --broad for PolII using matched inpt DNA as a control. ATAC seq peak was called with MACS with -q 0.05, --nomodel and --nolambda.

Data quality: Quality control of the reads was performed with FastQC v0.74, MultiQC v1.27.

Software: Reads were aligned to the mouse reference genome (mm10) using the Bowtie2 v2.4.2. PCR duplicates were removed using Picard. SAMtools v1.11 was used to sort BAM files and was converted to bigwig files by deepTools v3.5.4. The peaks were called with MACS2 v2.1.0. Differential ATAC seq or Cut&Tag peaks were identified by DiffBind v3.12.0. Data were annotated and visualized using bioconductor-CHIPseeke v1.28.3 and IGV v.2.11.9.

Flow Cytometry

Plots

Confirm that:

- [x] The axis labels state the marker and fluorochrome used (e.g. CD4-FITC).
- [x] The axis scales are clearly visible. Include numbers along axes only for bottom left plot of group (a 'group' is an analysis of identical markers).
- [x] All plots are contour plots with outliers or pseudocolor plots.
- [x] A numerical value for number of cells or percentage (with statistics) is provided.

Methodology

Sample preparation: The cells were collected from central neuro system, spleen, lymph nodes, lung, and colon, filtered with strainer, stained and analyzed for flow cytometry. For the preparation of single-cell suspension from CNS and spleen, lung, and colon, red blood cells were lysed by Red Blood Cell Lysis buffer.
Lung cells were prepared by type IV collagenase digestion and enriched using a 40% Percoll gradient.
The central nervous system tissues were finely minced before being mechanically dissociated through a 70 µm cell strainer. The resulting cell suspensions were enriched using a 40% Percoll gradient.
Colon cells were digested in RPMI 1640 medium containing DNase I and type III collagenase at 37°C for 20-30 min. Then the resulting cell suspensions were enriched using a 40% Percoll gradient.

Instrument: BD Fortessa 5 laser, BD AriaIII

Software: BD Diva, FlowJo V10

Cell population abundance: 95%-97% purity

Gating strategy:
effector-like CD4+ T cells (CD4+CD44hiCD62L–)
microglias (CD45mid CD11bhi)
myeloid cells (CD45hi CD11bhi)
eosinophils (CD45hi CD11bhi Siglec F+ CD11c-)
neutrophils (CD45hi CD11bhi Ly6G+ CD11c-)
naive CD4+ T (CD4+CD25–CD62LhiCD44low)
Th2 and Th17 cell gating strategy was described in the manuscript.

- [x] Tick this box to confirm that a figure exemplifying the gating strategy is provided in the Supplementary Information.
